# MiR-183-5p Induced by Saturated Fatty Acids Hinders Insulin Signaling by Downregulating IRS-1 in Hepatocytes

**DOI:** 10.3390/ijms23062979

**Published:** 2022-03-10

**Authors:** Mai Thi Nguyen, Kyung-Ho Min, Wan Lee

**Affiliations:** 1 Department of Biochemistry, College of Medicine, Dongguk University, 123 Dongdae-ro, Gyeongju 38066, Korea; nguyenmainhp@gmail.com (M.T.N.); dbdlaeo112@naver.com (K.-H.M.); 2 Channelopathy Research Center, College of Medicine, Dongguk University, 32 Dongguk-ro, Ilsan Dong-gu, Goyang 10326, Korea

**Keywords:** miR-183-5p, IRS-1, insulin resistance, obesity

## Abstract

Excessive saturated fatty acids (SFA) uptake is known to be a primary cause of obesity, a widely acknowledged risk factor of insulin resistance and type 2 diabetes. Although specific microRNAs (miRNAs) targeting insulin signaling intermediates are dysregulated by SFA, their effects on insulin signaling and sensitivity are largely unknown. Here, we investigated the role of SFA-induced miR-183-5p in the regulation of proximal insulin signaling molecules and the development of hepatic insulin resistance. HepG2 hepatocytes treated with palmitate and the livers of high-fat diet (HFD)-fed mice exhibited impaired insulin signaling resulting from dramatic reductions in the protein expressions of insulin receptor (INSR) and insulin receptor substrate-1 (IRS-1). Differential expression analysis showed the level of miR-183-5p, which tentatively targets the 3′UTR of *IRS-1*, was significantly elevated in palmitate-treated HepG2 hepatocytes and the livers of HFD-fed mice. Dual-luciferase analysis showed miR-183-5p bound directly to the 3′UTR of *IRS-1* and reduced IRS-1 expression at the post-transcriptional stage. Moreover, transfection of HepG2 hepatocytes with miR-183-5p mimic significantly inhibited IRS-1 expression and hindered insulin signaling, consequently inhibiting insulin-stimulated glycogen synthesis. Collectively, this study reveals a novel mechanism whereby miR-183-5p induction by SFA impairs insulin signaling and suggests miR-183-5p plays a crucial role in the pathogenesis of hepatic insulin resistance in the background of obesity.

## 1. Introduction

Excessive consumption of dietary saturated fatty acids (SFA) is closely associated with obesity and the ectopic accumulation of lipids, which can provoke lipotoxicity, metabolic dysfunction, and apoptosis in tissues [1,2]. As the liver is a vital organ in whole-body metabolic homeostasis, lipid burden in hepatocytes reduces insulin sensitivity and can lead to metabolic diseases, such as hepatic insulin resistance and type 2 diabetes (T2DM) [3]. The insulin signaling pathway is triggered when insulin binds to cell surface insulin receptor (INSR), and this interaction is followed by the phosphorylations of various proximal signaling intermediates, including insulin receptor substrate (IRS) and v-akt murine thymoma viral oncogene (Akt), and subsequently modulates various metabolic functions [4,5]. Hepatic insulin resistance is characterized by insufficient insulin sensitivity or impaired insulin signaling in the liver, which increases inappropriate gluconeogenesis and glucose output, consequently developing hyperglycemia and impeding whole-body energy utilization [6].

Although the etiology of hepatic insulin resistance in obesity is complicated and not fully understood, research over the last decade has demonstrated how excessive SFA intake can impair insulin signaling and result in T2DM [3,6]. In particular, palmitate, the most abundant SFA, increases the productions of diacylglycerol and ceramide, which can cause insulin resistance in several ways. Palmitate has been suggested to exacerbate oxidative stress and initiate the protein kinase C (PKC)/nuclear factor kappa B (NF-κB)/c-Jun N-terminal kinase (JNK)/mammalian target of rapamycin (mTOR) axis in hepatocytes [7]. Moreover, this signaling pathway, together with elevated diacylglycerol and ceramides, increases serine phosphorylation of IRS-1, inhibiting proximal insulin signaling and eventually leading to IRS-1 degradation [8]. IRS-1 belongs to the insulin receptor substrate family and plays a pivotal role in insulin signaling, glucose metabolism, and insulin-dependent mitogenesis in many cell types [9]. Although post-translational modification of IRS-1 is crucial for the regulation of insulin sensitivity, a number of studies have found that low IRS-1 protein levels are closely associated with the development of insulin resistance and T2DM [10,11]. IRS-1 protein levels were reported to be diminished in the liver of genetically modified obese animal models of T2DM, such as *ob*/*ob* mice [11,12] and Zucker rats [13]. In addition, IRS-1 expression has been known to be reduced in diabetic patients [14,15], and its depletion in mice resulted in insulin resistance and T2DM [4,9]. Furthermore, accumulating recent evidence has suggested that certain microRNAs (miRNAs) dysregulated in obesity are causally linked to hepatic insulin resistance by targeting IRS-1 [16,17,18].

MiRNAs are endogenous small non-coding RNAs with 21–25 nucleotides that play essential roles in post-transcriptional gene expression by binding to the 3′ untranslated regions (3′UTRs) of their target mRNAs [19]. Though the molecular targets and functions of individual miRNAs are not well understood, it has been reported that miRNAs regulate a variety of cellular processes, such as cell differentiation, proliferation, and metabolism [20]. Accordingly, dysregulations of miRNA expressions are associated with the etiologies of many diseases, including metabolic diseases, neurodegenerative diseases, and cancer [21]. In the last few decades, there has been increasing interest in the implications of specific miRNAs in metabolic regulation, such as glucose and lipid metabolism, whose derangement is implicated in insulin resistance and T2DM [22]. For example, miRNA dysregulations inhibit insulin signaling, pancreatic β-cell function, and critical metabolic pathways, including glycogen synthesis and gluconeogenesis [23]. However, the mechanisms connecting miRNA induced by SFA or obesity to hepatic insulin resistance are not well understood.

In this study, we hypothesized that specific miRNAs induced by SFA might be causally linked to impaired insulin signaling by suppressing the expression of proximal insulin signaling molecules. We investigated the insulin signaling pathway and miR-183-5p expressions in the liver of high-fat diet (HFD)-fed mice and palmitate-treated HepG2 hepatocytes. Moreover, we analyzed the direct targeting of miR-183-5p on IRS-1 3′UTR and demonstrated the detrimental effects of miR-183-5p on the insulin signaling pathway and insulin-stimulated glycogen synthesis.

## 2. Results

### 2.1. HFD Impaired Insulin Signaling and Elevated miR-183-5p Expression in the Liver of Mice

**Figure 1:**
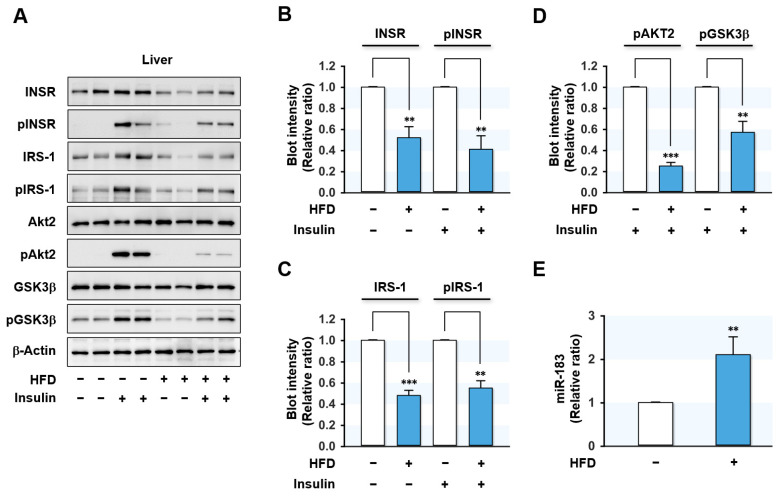
HFD led to impairment of insulin signaling and induced miR-183-5p in the mouse liver. C57BL/6N male mice were fed either NFD or HFD at the age of 6 wk for 14 wks. Mice were injected intraperitoneally with either insulin (1U/kg body wt) or the vehicle for the last 30 min prior to euthanasia. (**A**) Representative immunoblots (2 from 8 mice/group) of insulin signaling molecules (INSR, IRS-1, Akt2, and GSK3β) and their phosphorylations (pINSR, pIRS-1, pAkt2, and pGSK3β) in the liver are shown. (**B**–**D**) The respective densitometry measurements, normalized versus β-Actin. (**E**) The miR-183-5p levels from the liver of HFD-fed mice (closed column) and NFD-fed mice (open column) were determined using *q*RT-PCR. The results in immunoblots and *q*RT-PCR are presented as a relative ratio, normalized to NFD controls set to one. Results are presented as means ± SEMs. **, *p* < 0.01; ***, *p* < 0.001 vs. controls (NFD).

Since several miRNAs are known to participate in hepatic insulin resistance [16,17,24,25,26,27,28,29], we sought to identify the miRNAs upregulated by HFD in the liver of mice. Feeding the HFD for 14 wks dramatically increased mouse body weights and caused hyperglycemia, impaired oral glucose tolerance, and impaired insulin tolerance (Supplementary Figure S1), which are the hallmarks of insulin resistance and T2DM. Under this experimental condition, the expressions of proximal insulin signaling molecules, i.e., INSR and IRS-1 proteins, were significantly reduced in the liver of HFD-fed mice, whereas Akt2 and glycogen synthase kinase 3 beta (GSK3β) levels were unaffected (Figure 1A–C).

Next, we investigated the effects of HFD on the hepatic insulin signaling pathway by analyzing the phosphorylations of proximal insulin signaling molecules. Insulin-stimulated phosphorylations of INSR, IRS-1, Akt2, and GSK3β were markedly reduced in the liver of HFD-fed mice (Figure 1A–D), indicating that HFD-induced obesity impaired hepatic insulin signaling.

In our previous study, many miRNAs were elevated more than 1.5-fold in the liver of HFD-fed mice and palmitate-treated hepatocytes based on an Affymetrix miRNA array, and IRS-1 was the most drastically reduced insulin signaling molecule in the liver of HFD-fed mice [30,31,32]. Therefore, we selected several miRNAs that presumably target IRS-1, such as miR-183-5p, miR-376-3p, and miR-455-5p, based on the in silico analysis (performed using TargetScan and PicTar). Confirmation of miRNA expressions by *q*RT-PCR showed that the level of miR-183-5p increased more than 2-fold in the liver of HFD-fed mice (Figure 1E) compared to normal chow-fed mice, and thus, miR-183-5p was chosen to study further investigation for the IRS-1 targeting and implication in hepatic insulin resistance.

### 2.2. Palmitate Inhibited Insulin Signaling and Induced miR-183-5p Expression in HepG2 Hepatocytes

**Figure 2:**
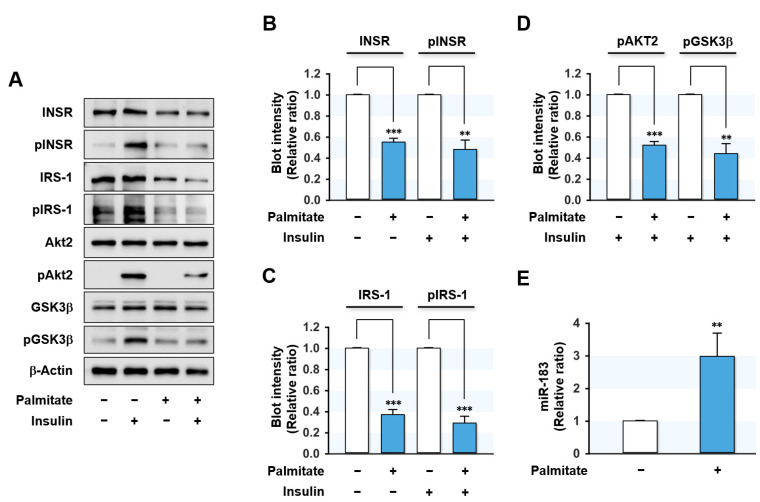
Palmitate impaired insulin signaling and elevated miR-183-5p expression in HepG2 cells. HepG2 cells were pretreated with BSA (control) or palmitate (0.5 mM) for 18 h and incubated with or without 100 nM insulin for the last 30 min. (**A**) Representative immunoblots of insulin signaling molecules (INSR, IRS-1, Akt2, and GSK3β) and their phosphorylations (pINSR, pIRS-1, pAkt2, and pGSK3β) are shown. (**B**–**D**) The respective densitometry measurements, normalized versus β-Actin. (**E**) The miR-183-5p levels were determined using *q*RT-PCR. The results in immunoblots and *q*RT-PCR are presented as a relative ratio, normalized to BSA controls set to one. Results are presented as means ± SEMs (*n* > 3). **, *p* < 0.01; ***, *p* < 0.001 vs. BSA controls.

Due to the correlation observed between the effects of HFD on the expressions of proximal insulin signaling molecules and miR-183-5p in the mice liver, we investigated whether miR-183-5p might be up-regulated in HepG2 hepatocytes treated with palmitate, the most abundant SFA in diet and plasma. We treated HepG2 cells with BSA (vehicle control) or BSA-conjugated palmitate (0.5 mM for 18 h) and evaluated its effects on insulin signaling and miRNA expressions. As shown in Figure 2A–D, palmitate significantly suppressed the protein expressions of proximal insulin signaling molecules, such as INSR and IRS-1, but did not affect the expressions of Akt2, GSK-3β, and β-Actin. Moreover, palmitate markedly inhibited the phosphorylations of INSR, IRS-1, and subsequent downstream molecules, including Akt2 and GSK-3β. Thus, these results indicate that palmitate impaired insulin signaling by downregulating the protein expressions and phosphorylation of INSR and IRS-1 in HepG2 hepatocytes. Interestingly, *q*RT-PCR showed miR-183-5p expression was significantly upregulated (approximately three-fold) by palmitate treatment (Figure 2). These results imply that the induction of miR-183-5p by SFA might be associated with the etiology of hepatic insulin resistance in obesity.

### 2.3. Palmitate Decreased IRS-1 and Upregulated miR-183-5p in a Dose- and Time-Dependent Manner

**Figure 3:**
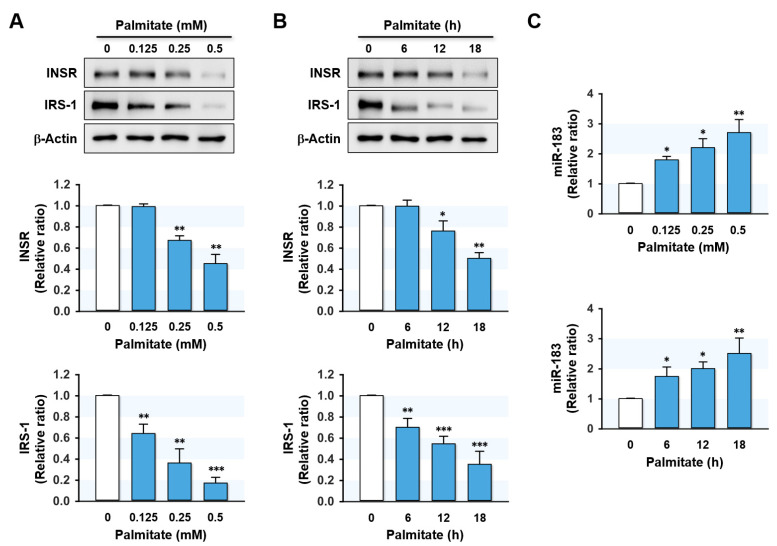
Palmitate regulated IRS-1 and miR-183-5p expressions in a dose- and time-dependent manner. HepG2 cells were preincubated with BSA or palmitate at 0.125–0.5 mM concentrations for 6 to 18 h. The expression levels of INSR and IRS-1 protein (**A**,**B**) or miR-183-5p (**C**) in various doses of palmitate and incubation time are shown. The protein expressions of INSR and IRS-1 were normalized versus β-Actin. The results in immunoblots and *q*RT-PCR are expressed as a relative ratio, normalized to control to one. Results are presented as means ± SEMs (*n* > 3). *, *p* < 0.05; **, *p* < 0.01; ***, *p* < 0.001 vs. BSA controls.

We next analyzed whether the expressions of INSR, IRS-1, and miR-183-5p were correlated dose- and time-dependently with palmitate treatment in hepatocytes. HepG2 cells were pretreated with BSA or palmitate at 0.125–0.5 mM concentrations for 6 to 18 h. Palmitate treatments remarkably and dose-dependently reduced INSR and IRS-1 protein levels as compared with controls (Figure 3A). Similarly, INSR and IRS-1 expression levels significantly decreased with increasing incubation time at a palmitate concentration of 0.5 mM (6–18 h) (Figure 3B). In contrast, it is interesting that cellular levels of miR-183-5p were upregulated dose- and time-dependently by palmitate (Figure 3C). These findings indicate that the levels of INSR and IRS-1 proteins are inversely correlated with the expression of miR-183-5p in palmitate-treated HepG2 hepatocytes.

### 2.4. MiR-183-5p Decreased IRS-1 Protein Expression by Directly Targeting the 3′UTR of IRS-1

**Figure 4:**
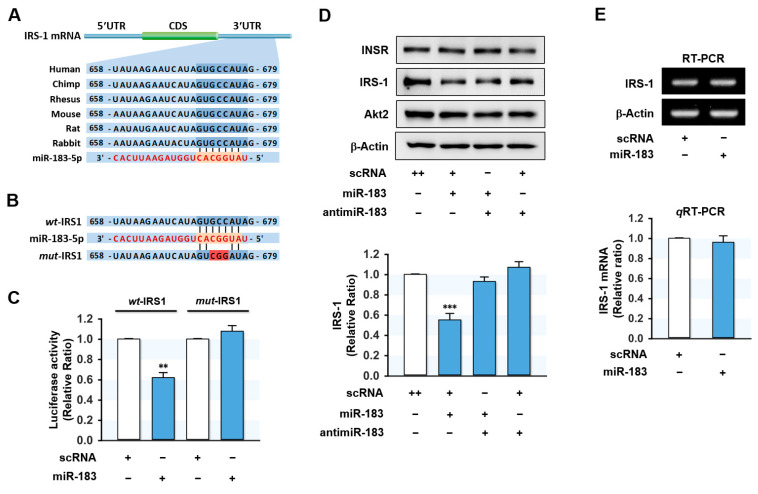
MiR-183-5p directly targeted the 3’UTR of IRS-1 and suppressed IRS-1 protein expression. (**A**) Graphic illustration of the conserved binding site for miR-183-5p within the 3′UTR of *IRS-1*. (**B**) The sequence of miR-183-5p binding site with wild-type (*wt*-IRS1) or mutant (*mut*-IRS1) 3′UTR of *IRS-1*. (**C**) The relative luciferase activity was performed after 24 h of reversed transfection with either the miR-183-5p or scRNA control (200 nM). (**D**) Representative immunoblots of INSR, IRS-1, and Akt2 after transfection with scRNA, miR-183-5p mimic, or antimiR-183 for 24 h. (**E**) Transcriptions of IRS-1 were determined by RT-PCR (upper) and *q*RT-PCR (lower). The results in immunoblots and *q*RT-PCR are expressed as a relative ratio, normalized to control set to one. Results are presented as means ± SEMs (*n* > 3). **, *p* < 0.01; ***, *p* < 0.001 vs. scRNA controls.

Since miR-183-5p expression was inversely related to INSR and IRS-1 levels in the HFD-fed mice liver and palmitate-treated hepatocytes, we next examined whether miR-183-5p directly regulates INSR and IRS-1 expressions by targeting their 3′UTRs. However, in silico target prediction analysis (TargetScan and PicTar) showed that a tentative binding site for the miR-183-5p seed sequence only existed in the 3′UTR of IRS-1 (Figure 4A) and not in that of INSR. Hence, we decided to confirm the direct binding between miR-183-5p and the 3′UTR of IRS-1 using a dual-luciferase reporter gene analysis. We produced a luciferase reporter construct containing an IRS-1 3’UTR segment of the wild-type (*wt*-IRS) or a binding site mutant (*mut*-IRS) of miR-183-5p in pmirGLO vector (Figure 4B). We then cotransfected the *wt*-IRS or *mut*-IRS reporter vector with scRNA or miR-183-5p mimic into HepG2 cells. As shown in Figure 4C, miR-183-5p mimic significantly reduced luciferase activity containing *wt*-IRS as compared with the scRNA control. Meanwhile, mutations in the tentative miR-183-5p binding site in the 3′UTR of IRS-1 (*mut*-IRS) almost completely abolished the inhibitory effect of miR-183-5p on luciferase activity. Thus, this result confirmed IRS-1 as a direct target of miR-183-5p.

To investigate the suppressive effect of miR-183-5p on IRS-1 expression further, HepG2 cells were transfected with miR-183-5p mimic or scRNA, and the expression levels of INSR and IRS-1 were determined. As expected, miR-183-5p mimic significantly reduced the protein expression of IRS-1, whereas the protein levels of INSR and Akt2 were unchanged (Figure 4D). Furthermore, cotransfection with antimiR-183 (a miR-183-5p inhibitor) almost entirely abolished the inhibitory effect of miR-183-5p mimic on the expression of IRS-1 (Figure 4D). On the other hand, IRS-1 mRNA levels were unaffected by miR-183-5p mimic transfection as determined by RT-PCR and *q*RT-PCR (Figure 4E), indicating that miR-183-5p downregulates IRS-1 expression at the post-transcriptional stage.

### 2.5. MiR-183-5p Mimic Hindered Insulin Signaling and Glycogen Synthesis

**Figure 5:**
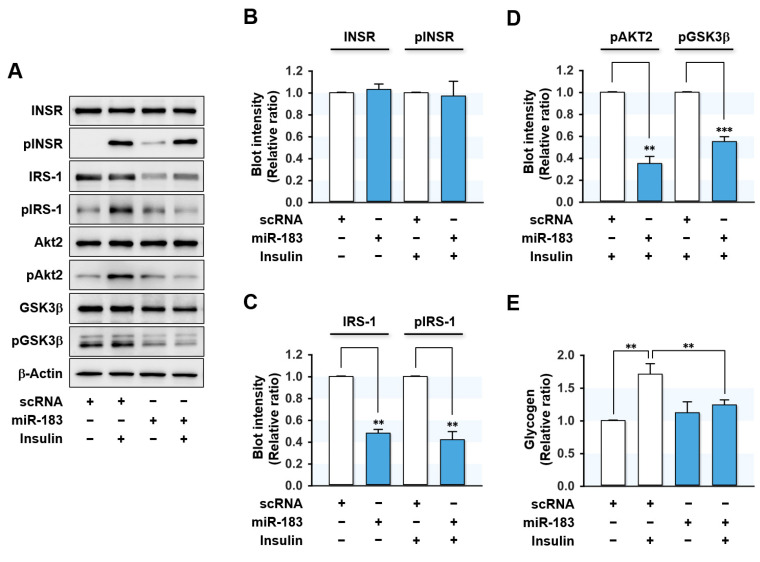
MiR-183-5p inhibited insulin signaling and insulin-stimulated glycogen synthesis. HepG2 cells were reverse transfected with 200 nM of scRNA or miR-183-5p mimic for 24 h and incubated additionally in the presence or absence of 100 nM insulin for the last 30 min before biochemical analysis. (**A**) Representative immunoblots of insulin signaling molecules (INSR, IRS-1, Akt2, and GSK3β) and their phosphorylations (pINSR, pIRS-1, pAkt2, and pGSK3β) are shown. (**B**–**D**) The respective densitometry measurements, normalized versus β-Actin. (**E**) The results from glycogen assays are displayed as relative ratios against the basal (without insulin stimulation) scRNA controls. Results are presented as means ± SEMs (*n* > 3). **, *p* < 0.01; ***, *p* < 0.001 vs. scRNA controls.

As miR-183-5p suppressed IRS-1 protein expression, we next investigated whether miR-183-5p induction provoked impaired insulin signaling in HepG2 hepatocytes. Transfection with miR-183-5p mimic drastically reduced IRS-1 expression and concomitantly inhibited insulin-stimulated IRS-1 phosphorylation without altering the expression or phosphorylation of INSR (Figure 5A,B). Furthermore, insulin-stimulated phosphorylations of downstream signaling proteins of IRS-1, such as Akt2 and GSK3β, were also significantly impeded by miR-183-5p mimic transfection (Figure 5A–D). This impaired proximal insulin signaling cascade was shown to be mainly ascribed to IRS-1 reduction. Next, we analyzed the effect of miR-183-5p on glycogen synthesis in HepG2 cells (Figure 5E). Insulin increased glycogen synthesis in scRNA control cells, whereas transfection with miR-183-5p mimic significantly decreased insulin-stimulated glycogen synthesis. Collectively, these data suggested that miR-183-5p hinders insulin signaling and insulin-stimulated glycogen synthesis by suppressing IRS-1 protein expression.

## 3. Discussion

Despite the impressive progress made in non-coding RNA biology, the mechanism whereby obesity-induced miRNAs are linked to the etiology of hepatic insulin resistance and T2DM is poorly understood. The present study has made the following significant advances to current knowledge: (i) Palmitate or HFD elevates miR-183-5p expression in hepatocytes. (ii) MiR-183-5p targets the 3′UTR of *IRS-1* mRNA directly and consequently suppresses IRS-1 expression at the post-transcriptional level. (iii) MiR-183-5p overexpression hinders insulin signaling and thus suppresses insulin-stimulated glycogen synthesis in HepG2 hepatocytes. This study reveals the crucial role of miR-183-5p in the insulin signaling pathway by targeting IRS-1 and suggests a novel mechanism for hepatic insulin resistance in obesity.

Hsa-miR-183-5p, located on human chromosome 7q32.2, is a member of the miR-183 family gene cluster and plays essential roles in diverse physiological and pathological processes, including metabolism, cell survival, and immunity [33]. The upregulation of miR-183-5p has been reported in a wide range of malignancies, including hepatocellular carcinoma [34], colon cancer [35], and breast cancer [36], although it has been reported to be downregulated in osteosarcoma [37] and endometriosis [38]. This diversity in expression profiles may be due to the cell type-specific differences in biochemical compositions and cellular regulation patterns. Nevertheless, our study shows an association between the upregulation of miR-183-5p and high SFA intake as a novel risk factor of hepatic insulin resistance. MiR-183-5p was significantly elevated in the liver of HFD-fed mice (Figure 1). In addition, miR-181-5p levels were found to increase in a time- and dose-dependent manner in palmitate-treated HepG2 cells (Figure 3). These results are consistent with those of a recent study, in which miR-183-5p was found to be upregulated in the livers of obese animal models, such as *ob*/*ob* and *db*/*db* mice [39]. Conversely, overexpression of miR-183-5p increased triglyceride accumulation and the transcriptions of lipogenic genes in hepatocytes, whereas miR-183-5p inhibition improved hepatic TG accumulation [39]. Furthermore, it was suggested that miR-183-5p might be a potential indicator of hepatic injury or inflammation [40]. From this point of view, it is interesting that obesity has been previously recognized as chronic inflammation, which is causally linked to insulin resistance and T2DM [41]. Considering that miR-183-5p is inducible in obesity and inflammation and promotes fat accumulation, miR-183-5p may play a pivotal role in the vicious cycle of obesity, metabolic disease, and chronic inflammation.

Notably, miR-183-5p induction suppressed IRS-1 expression at the post-transcriptional level, thereby hindering proximal insulin signaling in HepG2 hepatocytes. Although serine phosphorylations of IRS-1 constitute a well-established mechanism for insulin resistance, several studies have demonstrated that reductions in IRS-1 protein levels are also a significant risk factor for insulin resistance and T2DM [4,9,10]. Physiologically, various mechanisms are responsible for the regulation of IRS-1 expression. At the transcriptional level, IRS-1 expression is regulated by various regulators, including the transcriptional repressor AP2β and the nuclear receptor coactivators p300/CBP interacting protein (p/CIP) and steroid receptor coactivator 1 (SRC1) [42,43]. In the post-translational stage, IRS-1 levels depend on ubiquitin-mediated degradation associated with serine/threonine phosphorylation of IRS-1 [44,45]. Furthermore, IRS-1 undergoes ubiquitinylation and proteasomal degradation mediated by suppressor of cytokine signaling (SOCS) in the presence of nutrient excess, inflammation, or hyperinsulinemic conditions [46,47]. Interestingly, SFA is also known to inhibit insulin signaling and to promote the ubiquitination of key signaling molecules, such as IRS-1 and Akt [48]. During the last decade, it has been reported that various miRNAs induced by SFA participate in the development of hepatic insulin resistance by targeting the translation of proximal insulin signaling molecules at the post-transcriptional level. For example, miR-15b, miR-96, miR-195, and miR-424–5p were upregulated in the livers of HFD-fed mice, and these upregulations were associated with INSR downregulation [24,25,26,27]. Likewise, IRS-1 was found to be targeted by miR-222 and miR-145 in the liver and adipose tissues [16,17]. Our findings suggest that the induction of miR-183-5p by SFA is detrimental to insulin signaling as it impairs IRS-1/Akt/GSK-3β axis. Although SFA has been shown to downregulate the expressions of INSR and IRS-1 via multiple mechanisms, this study demonstrates that SFA-inducible miR-183-5p contributes to IRS-1 reduction in hepatocytes and suggests that induction of miR-183-5p contributes toward hepatic insulin resistance in a background of obesity.

Then what is the mechanism underlying the upregulation of miR-183-5p by SFA or obesity? Although our understanding of the mechanism responsible for miR-183-5p expression is limited at this moment, target mRNA analysis exhibited that specific regions on the miR-183-5p promoter might contain highly conserved binding sites for transcription regulators associated with obesity, such as peroxisome proliferators-activated receptor gamma (PPARγ) and CCAAT enhancer-binding protein alpha (C/EBPα). PPARγ is a member of the nuclear receptor superfamily and is involved in cell proliferation, growth, and differentiation [49]. Moreover, its activation is associated with lipid deposition in the liver via the induction of lipogenic genes [49]. Previously, PPARγ activation was reported to be a downstream target of transforming growth factor-beta (TGF-β)/SMAD family member 3 (SMAD3) signaling with the coactivation of C/EBPα, an adipogenic transcription factor, in HFD-induced obese mice [50]. Interestingly, plasma TGF-β levels have been reported to be elevated in obese humans, HFD-fed mice, and *ob*/*ob* mice [51]. In addition, lipid accumulation in the liver also activated TGF-β transcription in humans [52]. Therefore, these obesity-derived activations of PPARγ and C/EBPα in consort with TGF-β upregulation may constitute a mechanism for the upregulation of miR-183-5p in obesity [53]. Although additional study is warranted to elucidate the transcriptional modulators of miR-183-5p, previous results indicate miR-183-5p may be a crucial mediator of the association between obesity and hepatic insulin resistance.

## 4. Materials and Methods

### 4.1. Cell Culture and Palmitate Treatment

Human hepatocellular carcinoma cells (HepG2 cells; ATCC #77400, Manassas, VA, USA was maintained in Minimal Essential Medium (MEMα, Gibco, Carlsbad, CA, USA) containing 10% fetal bovine serum (FBS, Gibco) and 1% antibiotics (penicillin-streptomycin, Gibco) in a 5% CO_2_ atmosphere at 37 °C. Briefly, cells were grown on 35 mm plates at a seeding density of 5×10^5^ cells per well and allowed to grow until 30 to 40% confluent before being subjected to reverse transfection or treatments. BSA-conjugated palmitate solution was prepared for palmitate treatments, as previously described [27]. HepG2 cells were pretreated with BSA or palmitate (0–0.5 mM) for 6 to 18 h. When appropriate, they were subsequently incubated for 30 min with or without insulin (100 nM).

### 4.2. Transfection of Oligonucleotides

Oligonucleotides, including scrambled control RNA (scRNA), miR-183-5p mimic, and antimir-183-5p (a 2′-O-methyl-modified antisense oligonucleotide against mature miR-183-5p) were purchased from Genolution (Seoul, Korea). HepG2 cells were transfected with the 200 nM of oligonucleotides using G-fectin (Genolution), according to the manufacturer’s instructions. For luciferase activity assays, cells (5 × 10^4^ cells) were grown in 12-well plates for 24 h and then cotransfected either scRNA or miR-183-5p (200 nM) and plasmid (100 mg) containing the targeted gene fragment using Lipofectamine 2000 (Invitrogen, Waltham, MA, USA). Oligonucleotide sequences are provided in Table S1.

### 4.3. RNA Preparation and Quantitative Real-Time RT-PCR (qRT-PCR)

Total RNAs from liver tissues or HepG2 cells were isolated and purified using Qiazol reagent and the miRNeasy Mini Kit (Qiagen, Hilden, Germany). RNA concentrations and qualities were confirmed by spectrophotometry (UV-1700 PharmaSpec; Shimadzu, Japan) and gel electrophoresis. cDNAs were synthesized using the miScript II RT Kit (Qiagen). To determine mRNA and miRNA expression levels, *q*RT-PCR was performed in a LightCycler 480 (Roche-Applied Science, Mannheim, Germany) using SYBR Green I and iTaq polymerase (Promega, Madison, WI, USA). Quantification was performed using the 2^−ΔΔCt^ method, and U6 was used as the internal control. Primer sequences and reaction conditions are summarized in Table S2.

### 4.4. Dual-Luciferase Assay

The segment of *IRS-1* 3′UTR (252 nt, *wt*-IRS1) containing the miR-183-5p binding site was subcloned into the pmirGLO vector (Promega) to produce a wild-type plasmid. Site-directed mutagenesis was conducted using overlapping oligonucleotides without the miR-183-3p-binding region (*mut*-IRS1) to generate a mutant plasmid. The primers sequences used for subcloning and mutagenesis are listed in Table S2. Luciferase assays were conducted using the Dual-Luciferase Reporter Assay System kit (Abcam, Cambridge, UK), as previously described [28]. The cells were seeded on a 12-well plate, and 24 h later, wild-type or mutant plasmid were cotransfected with miR-183-5p or scRNA and then homologized using lysis buffer after 24 h of transfection (Promega). Luciferase activities were assayed using a Sirius L luminometer (Titertek-Berthold, Pforzheim, Germany). Relative luciferase activity was defined as the percentage of cells exhibiting Firefly out of Renilla luminescence.

### 4.5. Immunoblot Analysis

HepG2 cells were lysed in PBS containing 0.2 mM phosphatase inhibitor cocktail 2 (Sigma, Ronkonkoma, NY, USA), 2% Triton X-100, and 1% PMSF. Total protein concentrations were analyzed using the Bradford assay (Bio-Rad). Proteins (20 µg/lane) were resolved by SDS-PAGE and blotted onto nitrocellulose membranes (Amersham Biosciences, Piscataway, NJ, USA), which were blocked with non-fat milk (5%) (Becton, France) for 1 h, and then incubated overnight with specific antibodies at 4 °C (as described in Table S3), washed with TTBS (Tween 20-TBS), and developed with secondary antibodies. Images were obtained using Evolution Capt software (Vilber, France) and commercial Femto reagent (Thermo Fisher Scientific, Waltham, MA, USA). Quantification was performed using Evolution Capt software (Vilber, France).

### 4.6. Glycogen Assay

The colorimetric assay Kit II (Biovision, CA, USA) was used to determine glycogen levels. Briefly, miR-183-5p or scRNA was reverse transfected into HepG2 cells in FBS-free MEMα for 4 h. The medium was then replaced with free DMEM (without FBS) (Gibco) and incubated for 2 h with or without insulin (100 nM). The next day, cells were harvested, lysed, and lysates were mixed with glycogen hydrolysis buffer and hydrolysis enzyme and incubated for 30 min. Absorbances were measured at 450 nm using a microplate reader (Model 680; Bio-Rad, Hercules, CA, USA).

### 4.7. Animal Experiment

Acclimated 6 wks-old C57BL/6N male mice (OrientBio, Gyeonggi, Korea) were randomly divided into two groups: a high fat diet group (HFD group; 60% energy from fat; Dyets Inc., Bethlehem, PA, USA) or a normal fat diet (NFD group, 11% energy from fat; Purina, Wilkes-Barre, PA, USA). The detailed compositions of the two diets are shown in Table S4. The mice (8 mice/group) were allowed access to water and food ad libitum. After being fed the HFD or NFD for 14 weeks, oral glucose tolerance testing (OGTT) and insulin tolerance testing (ITT) were performed as described in Supplementary Figure S1. Three days after ITT, the mice were fasted overnight, and insulin (1U/kg body weight, Sigma-Aldrich) was administered in the last 30 min prior to euthanasia; the mice were then subjected to biochemical analysis. The in vivo experimental protocol was approved beforehand by the Animal Use and Care Committee of Dongguk University (approval IACUC-2020-007).

### 4.8. In Silico Analysis and Statistical Analysis

The binding site of miR-183-5p on IRS-1 3’UTR was predicted by publicly available algorithms (TargetScan: www.targetscan.org, Pictar: pictar.mdc-berlin.de). Results are expressed as the means ± SEMs of at least three independent experiments. The statistical analysis was performed using the Student’s *t*-test for unpaired data.

## 5. Conclusions

This study reveals the crucial role of miR-183-5p in the insulin signaling pathway by targeting IRS-1. MiR-183-5p was induced by SFA palmitate and suppressed IRS-1 expression by targeting the 3′UTR of *IRS-1,* and the ectopic expression of miR-183-5p mimic suppressed IRS-1 expression, consequently inhibiting proximal insulin signaling and insulin-stimulated glycogen synthesis in HepG2 hepatocytes. Therefore, the current study suggests that miR-183-5p upregulated by SFA or obesity contributes to the development of hepatic insulin resistance and T2DM by suppressing IRS-1.

## Data Availability

The data presented in this study are available on request from the corresponding author.

## Supplementary Materials

The following supporting information can be downloaded at: https://www.mdpi.com/article/10.3390/ijms23062979/s1.
