# Supplementary material for: MiR-183-5p Induced by Saturated Fatty Acids Hinders Insulin Signaling by Downregulating IRS-1 in Hepatocytes
Source: Int J Mol Sci. 2022 Mar 10;23(6):2979. doi: 10.3390/ijms23062979 (PMC8953084; doi:10.3390/ijms23062979)
Supplement: Supplementary file 1 [file ijms-23-02979-s001.zip › Supplementary Figure S1.pdf]

Supplementary Figure S1

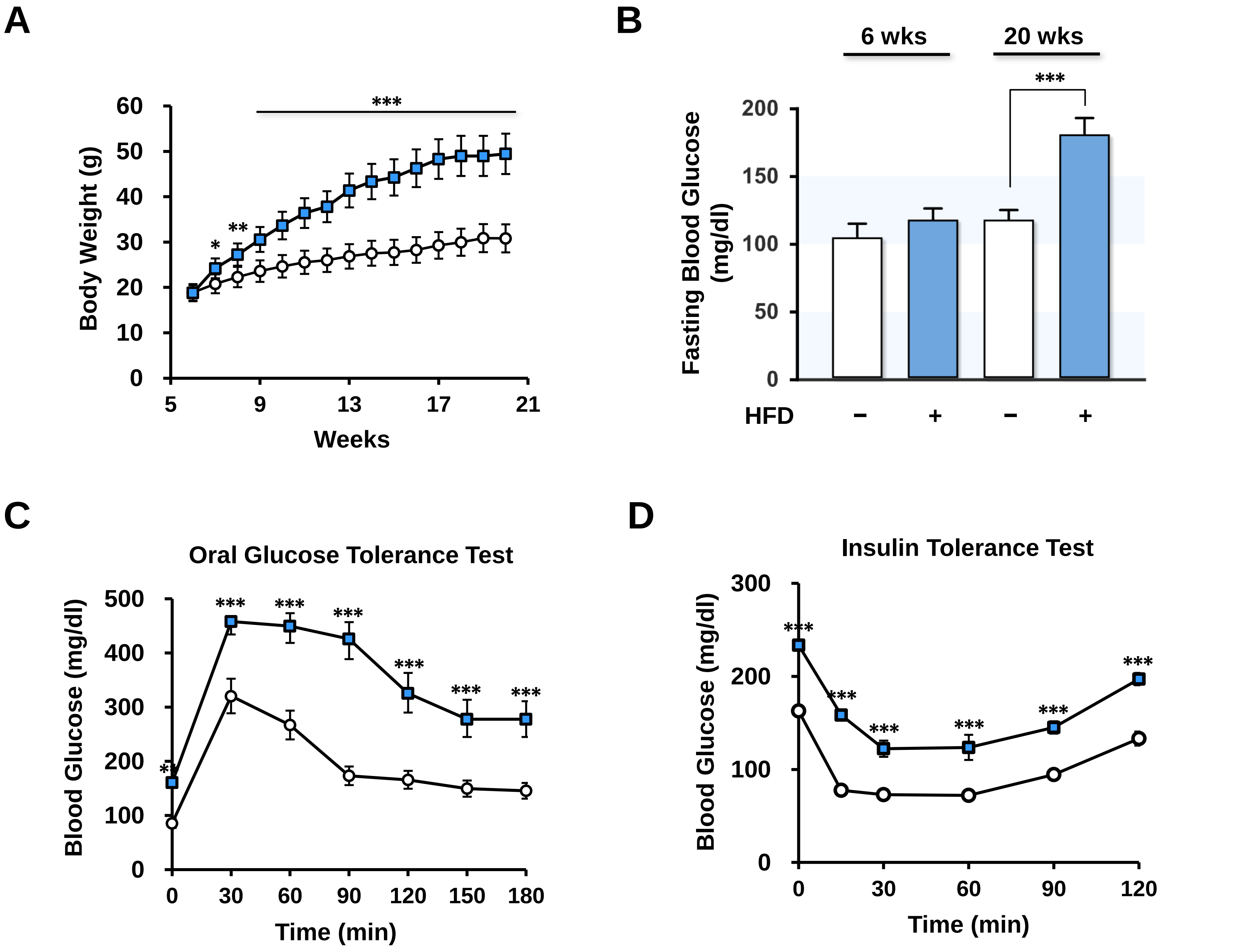

Supplementary Figure S1. HFD induced hyperglycemia, impaired glucose tolerance, and impaired insulin tolerance in mice.

For the diet-induced obese mice model, C57BL/6N male mice were fed either NFD or HFD at the age of 6 wk for 14 wks, as described in the Method section.

(A) The body weight was measured every week. HFD increased body weights significantly after 1 wk of feeding. **(B)** After 14 wks of HFD feeding (at the age of 21 wk), fasting blood glucose level was significantly increased in mice, indicating that HFD provoked hyperglycemia in mice. **(C)** For the oral glucose tolerance test (OGTT), mice were fasted overnight, and 20 % glucose solution (10 ml/kg of body weight) was administered orally with an 18-gauge gavage needle. Blood was drawn from a tail vein at 0, 30, 60, 120, and 180 min, and plasma glucose concentrations were measured with the Blood Glucose Monitoring System (SD Biosensor, Seoul, Korea). **(D)** For the insulin tolerance test (ITT), the mice were fasted 3 h, and insulin (1 U/kg of body weight) was injected intraperitoneally. Blood was drawn from a tail vein at 0, 15, 30, 60, 90, and 120 min, and the blood glucose concentrations were measured.

The whole-body disposal of blood glucose was significantly delayed in the HFD-fed mice compared to NFD-fed mice, suggesting that HFD caused hyperglycemia, IGT, and ITT in mice.

Data are expressed as mean ± SEM. from 8 mice, for NFD control (open circles or open column) and HFD (blue rectanglular or blue column); * $P < 0.05$; ** $P < 0.01$; *** $P < 0.001$ vs. NFD control.
