# Supplementary material for: MiR-183-5p Induced by Saturated Fatty Acids Hinders Insulin Signaling by Downregulating IRS-1 in Hepatocytes
Source: Int J Mol Sci. 2022 Mar 10;23(6):2979. doi: 10.3390/ijms23062979 (PMC8953084; doi:10.3390/ijms23062979)
Supplement: Supplementary file 1 [file ijms-23-02979-s001.zip › Table S1-S4.pdf]

Table S1. Oligonucleotide sequences for transfection

Gene	Primer sequence (5´-3´)
scRNA (control RNA)	UCACAACCUCCUAGAAAGAGUAGA
miR-183-5p	UAUGGCACUGGUAGAAUUCACU
antimiR-183	AGUGAAUUCUACCAGUGGATG

Table S2. Primer lists and conditions for *q*RT-PCR, RT-PCR, and cloning

(A) Human primer lists for *q*RT-PCR and RT-PCR

Gene	Primer sequence (5′-3′)		Product size	Annealing Temperature	Concentration		Cycle
					cDNA	Primer	
miR-183-5p	F.P	TATGGCACTGGTAGAATTCACT	90	55	2 ng/μl	0.5 μM	40
miRNA universal Primer	R.P	miScript universal primer (Qiagen)					
IRS1	F.P	GTTTCCAGAAGCAGCCAGAG	474	60			
	R.P	ACTCTCTCCACCCAACGTGA					
U6	F.P	CTCGCTTCGGCAGCACA	94	58			
	R.P	AACGCTTCACGAATTTGCGT					
β-Actin	F.P	TCACCCACACTGTGCCCATCTACGA	348	58			
	R.P	GGATGCCACAGGATTCCATACCCA					

(B) Primer lists for wild-type and mutant 3'UTR cloning

Gene	Primer sequence (5′-3′)		Product size	Annealing Temperature	Concentration		Cycle
					cDNA	Primer	
wt-IRS1	F.P	TTGGATGTGGGATGGAGGTA	275	58	2 ng/μl	0.5 μM	35
	R.P	AACATGGCCTATTGTCTTAC					
mut-IRS1	F.P	GAATCATAGTCGGATAGATGG	146				
	R.P	AACATGGCCTATTGTCTTAC					
	F.P	TTGGATGTGGGATGGAGGTA	150				
	R.P	CCATCTATCCGACTATGATTC					

Table S3. Antibodies list

Antibody	Type	Targeted species	Manufacturer	Cat. No.	Dilution ratio*
IRS-1	Polyclonal	Rabbit	Millipore, Burlington, MA, USA	#06-248	1:5,000
Phospho IRS-1(Tyr612)	Polyclonal	Rabbit	Invitrogen, Thermofisher Scientific, Waltham, MA, USA	Sc250575	1:2,000
INSR	Monoclonal	Mouse	Cell Signaling Technology, Danvers, MA, USA	#3020	1:3,000
Phospho INSR (Tyr1150/1151)	Monoclonal	Rabbit	Cell Signaling Technology, Danvers, MA, USA	#3024	1:3,000
Akt2	Polyclonal	Rabbit	Cell Signaling Technology, Danvers, MA, USA	#9272	1:10,000
Phospho Akt2 (Ser474)	Polyclonal	Rabbit	Cell Signaling Technology, Danvers, MA, USA	#8599	1:10,000
GSK3β	Monoclonal	Rabbit	Cell Signaling Technology, Danvers, MA, USA	#9315	1:10,000
Phospho GSK3β (Ser21/9)	Polyclonal	Rabbit	Cell Signaling Technology, Danvers, MA, USA	#9331	1:10,000
β-actin	Monoclonal	Rabbit	Sigma-Aldrich Chemical, St. Louis, Missouri, USA	A2066	1:10,000
Antibodies HRP-linked anti-rabbit IgG			Cell Signaling Technology, Danvers, MA, USA	#7074	1:10,000
Goat anti-mouse(H+L)			Invitrogen, Thermofisher Scientific, Waltham, MA, USA	#32430	1:2,000

*All blots were visualized using a Femto reagent (Thermofisher Scientific).

Table S4. Diet compositions

(A) Normal fat diet (NFD; Purina Laboratory Rodent Diet 38057)

Calories (%)		Fat component of total fat (gram%)		
		Saturated fatty acid	Unsaturated Fatty acids	Unidentified fat
Fat	12.41	Arachidonic Acid (4.42)	Omega-3 fatty acid (24.55)<br>Linoleic acid (24.15)<br>Linolenic acid (2.65)	Unidentified (44.23)
Carbohydrate	63.07			
Protein	24.52			
Total	100	4.42 of total fat	51.35 of total fat	44.23 of total fat

(B) High-fat diet (HFD; D12492)

Calories (%)		Fat component of total fat (gram%)	
		Saturated fatty acid	Unsaturated Fatty acids
Fat	60	Palmitic acid (19.64)<br>Stearic acid (10.59)<br>Myristoleic acid (1.10)<br>Lau acid (0.8)<br>Margaric acid (0.35)<br>Arachidic acid (0.16)<br>Pentadecanoic acid (0.08)<br>Capric acid (0.04)	Oleic acid (33.68)<br>Linoleic acid (28.37)<br>Linolenic acid (2.04)<br>Arachidonic acid (1.78)<br>Palmitoleic acid (1.33)<br>Docosapentaenoic acid (0.78)
Carbohydrate	20		
Protein	20		
Total	100	32 of total fat	68 of total fat
